# Cytotoxicity, Intracellular Replication, and Contact-Dependent Pore Formation of Genotyped Environmental *Legionella pneumophila* Isolates from Hospital Water Systems in the West Bank, Palestine

**DOI:** 10.3390/pathogens10040417

**Published:** 2021-04-01

**Authors:** Ashraf R. Zayed, Marina Pecellin, Lina Jaber, Suha Butmeh, Shereen A. Bahader, Michael Steinert, Manfred G. Höfle, Ingrid Brettar, Dina M. Bitar

**Affiliations:** 1 Department of Vaccinology and Applied Microbiology, Helmholtz Centre for Infection Research (HZI), Inhoffenstrasse 7, 38124 Braunschweig, Germany; ashraf.rashadlab@gmail.com (A.R.Z.); marinapecellin@gmail.com (M.P.); manfred.hoefle@helmholtz-hzi.de (M.G.H.); ibrettar@web.de (I.B.); 2 Department of Microbiology and Immunology, Faculty of Medicine, Al-Quds University, Abu-Dies, East Jerusalem 9993100, Palestine; lina19486@hotmail.com (L.J.); salbutmeh@staff.alquds.edu (S.B.); Shereenbahader@hotmail.com (S.A.B.); 3 Institut für Mikrobiologie, Technische Universität Braunschweig, 38106 Braunschweig, Germany; m.steinert@tu-bs.de

**Keywords:** *Legionella pneumophila*, genotype, MLVA, clonal complex, Gt10(93), VACC11, virulence

## Abstract

*Legionella pneumophila* is the causative agent of Legionnaires’ disease. Due to the hot climate and intermittent water supply, the West Bank, Palestine, can be considered a high-risk area for this often fatal atypical pneumonia. *L. pneumophila* occurs in biofilms of natural and man-made freshwater environments, where it infects and replicates intracellularly within protozoa. To correlate the genetic diversity of the bacteria in the environment with their virulence properties for protozoan and mammalian host cells, 60 genotyped isolates from hospital water systems in the West Bank were analyzed. The *L. pneumophila* isolates were previously genotyped by high resolution Multi Locus Variable Number of Tandem Repeat Analysis (MLVA-8(12)) and sorted according to their relationship in clonal complexes (VACC). Strains of relevant genotypes and VACCs were compared according to their capacity to infect *Acanthamoeba castellanii* and THP-1 macrophages, and to mediate pore-forming cytotoxicity in sheep red blood cells (sRBCs). Based on a previous detailed analysis of the biogeographic distribution and abundance of the MLVA-8(12)-genotypes, the focus of the study was on the most abundant *L. pneumophila*- genotypes Gt4(17), Gt6 (18) and Gt10(93) and the four relevant clonal complexes [VACC1, VACC2, VACC5 and VACC11]. The highly abundant genotypes Gt4(17) and Gt6(18) are affiliated with VACC1 and sequence type (ST)1 (comprising *L. pneumophila* str. Paris), and displayed seroroup (Sg)1. Isolates of these two genotypes exhibited significantly higher virulence potentials compared to other genotypes and clonal complexes in the West Bank. Endemic for the West Bank was the clonal complex VACC11 (affiliated with ST461) represented by three relevant genotypes that all displayed Sg6. These genotypes unique for the West Bank showed a lower infectivity and cytotoxicity compared to all other clonal complexes and their affiliated genotypes. Interestingly, the *L. pneumophila* serotypes ST1 and ST461 were previously identified by in situ-sequence based typing (SBT) as main causative agents of Legionnaires’ disease (LD) in the West Bank at a comparable level. Overall, this study demonstrates the site-specific regional diversity of *L. pneumophila* genotypes in the West Bank and suggests that a combination of MLVA, cellular infection assays and hierarchical agglomerative cluster analysis allows an improved genotype-based risk assessment.

## 1. Introduction

*Legionella pneumophila*, the causative agent of legionellosis, inhabits natural and man-made freshwater environments [1,2,3]. The pathogen preferentially thrives in biofilm communities, where it infects and replicates within protozoan hosts [1,3]. Human infection with *L. pneumophila* occurs by inhaling contaminated aerosols [4,5] and affected patients develop either a severe atypical pneumonia known as Legionnaires’ disease (LD), or a minor flu-like illness called Pontiac fever [6,7,8,9,10].

During human infection *L. pneumophila* replicates intracellularly within alveolar macrophages and epithelial cells [11,12,13,14]. The involved pathogenicity mechanisms strongly resemble the infection of protozoan hosts [3,11]. In protozoa and human macrophages *L. pneumophila* induces the formation of a replicative-permissive membrane-bound compartment called “*Legionella*-containing vacuole” (LCV) [14,15,16,17]. 

The most important virulence mechanism of *L. pneumophila* relies on the delivery of more than 300 different effector proteins into host cells by the bacterial Dot (Defect in Organelle Trafficking)/Icm (intracellular multiplication) type IV secretion system (T4SS). The Dot/Icm effectors target many host cell processes, lead to the recruitment of mitochondria and ER-derived vesicles to LCVs and mediate evasion of the host’s degradative lysosomal pathway, enabling *L. pneumophila* to replicate [16,17,18,19,20,21,22]. Furthermore, the Dot/Icm system is essential for pore-formation mediated lysis of the host cell [12,17,19,22,23,24].

Since virulence of *L. pneumophila* is determined by factors encoded in the genome and by environmental drivers [9,25,26] a previous study performed at Haifa, Israel, strongly suggests a link between genotype and virulence of *L. pneumophila* strains [9]. However, the high diversity of environmental *L. pneumophila* strains and the lack of detailed insights in their ecology are regarded as a major problem for management and prevention measures of infections [27]. Many studies demonstrated that the main sources for LD are potable water systems in large buildings [10,26,28,29,30,31,32]. Especially contaminated hospital water systems pose a high risk since elderly and immunosuppressed people are highly susceptible to LD [33]. Thus, understanding *L. pneumophila* ecology and genetic polymorphism in hospitals may help to develop better health control protocols [34,35,36]. 

Differences in ecology and pathogenicity were already described for various *L. pneumophila* genotypes colonizing drinking water distribution systems (DWDSs) [33,37]. Multi locus variable number of tandem repeats (VNTR) analysis (MLVA) using 13 loci designated as MLVA-8(12) was successfully used to assess the genetic diversity among *L. pneumophila* isolates. VNTRs consist of relatively short DNA fragments repeated in tandem and can vary in copy number among strains [38,39,40,41,42]. Recent publications demonstrated that the majority of clinically relevant strains can be grouped into a limited number of Clonal Complexes (CCs) defined by MLVA, called VNTR analysis CC (VACC) [38,41]. MLVA can be used to complement recommended Sequence-Based Typing (SBT) and gain insights into the clonal structure of *L. pneumophila* populations. Many studies have used MLVA for the genotyping of *L. pneumophila* strains [38,39,40,41,42]. They showed the high correspondence between MLVA genotypes and STs with an important increase in resolution when applying MLVA, which is relevant for understanding clonal populations. Due to its high resolution power, MLVA could complement SBT for large sets of isolates and enable insights into the clonal structure of *L. pneumophila* populations, as well as helping in strain selection for more details by whole genome sequencing. It is of special relevance for the large and globally important ST1 (comprising *L. pneumophila* Paris), where a higher resolution is needed for clinical and source tracking issues. 

The current study on virulence traits is based on the results of two former studies [38,43]. In comprehensive two-year surveillance, DWDS of eight hospitals across the West Bank were analyzed with respect to *L. pneumophila* occurrence by culture and PCR, and environmental parameters. The retrieved 180 *L. pneumophila* isolates showed a high diversity of 27 MLVA-8(12) genotypes, affiliated to four clonal complexes (VACC 1; 2; 5; 11). The MLVA-8(12) genotypes showed a specific biogeographic pattern across the West Bank, with a high (20/27) fraction of genotypes unique for the West Bank. Most (18/27) of the genotypes were highly endemic in the West Bank. Most dominant were strains of VACC1 (ST1) comprising the ubiquitous and most abundant genotype Gt4(17) and the endemic Gt6(18). VACC11 (ST461) was the second largest clonal complex comprising three abundant genotypes (Gt10(93), Gt10(141), Gt9(92)). The major fraction of the strains was affiliated with ST1 and ST461 and Sg1 and Sg6, sequence types and serogroups of high clinical relevance in the West Bank [32].

A focus of the former study in the West Bank was the identification of environmental drivers influencing abundance and genotype composition. The study indicated a suppressive effect of high magnesium concentrations (>30 mg/L) on *L. pneumophila* abundance in water and biofilm in West Bank DWDS. Furthermore, according to their physico-chemical habitat conditions, the major genotypes were attributed to three different niches; three to four genotypes were sharing a common niche and were considered to represent a specific ecotype. Thus, environmental drivers exerted a different influence in response to the genotype [43,44].

Based on the previous studies investigating *L. pneumophila* abundance and genotype composition across the West Bank, we compared the virulence of 60 environmental isolates. Emphasis was on the characterization of three dominant MLVA-8(12) genotypes and the four clonal complexes occurring in the West Bank. For virulence assessment three in vitro tests were used: cytotoxicity assays against *Acanthamoeba castellanii* and THP1 macrophages, and pore-forming mediated cytotoxicity using sheep red blood cells (sRBCs).

## 2. Material and Methods 

### 2.1. L. pneumophila Isolates

This study included 60 *L. pneumophila* environmental strains isolated from eight hospitals in the West Bank, Palestine (Table 1 and Supplementary Material Table S1). Genotyping was conducted using multilocus variable number of tandem repeat analysis using 13 loci—MLVA-13 (MLVA-12 plus 1 loci unique for the MLVA-8 scheme) designated as (MLVA-8(12) [38,45] assigning the majority of the strains to three genotypes (Gt4(17), Gt6(18), and Gt10(93)) and four clonal complexes (VACC1, VACC2, VACC5 and VACC11) [38]. Table 1 describes the details of the environmental *L. pneumophila* isolates used for this study. As reference strains *L. pneumophila* Philadelphia-1 ATCC33152^T^ [46] and its Icm/Dot deficient *dotA* mutant [47,48] were used as positive and negative controls, respectively. Also, *L. pneumophila* str. Paris CIP107629 [49] was used as a positive control (Table 1). All tests of the strains were run as triplicates. A detailed protocol on the *L. pneumophila* inoculum preparation for the cytotoxicity tests was previously described [9].

### 2.2. Acanthamoeba castellanii Cytotoxicity Assay

The virulence of *L. pneumophila* isolates was measured by infecting *A. castellanii* (ATCC 50374) at multiplicity of infection (MOI) of 10 for 24 h as previously described [9,50]. The percentage of survived *A. castellanii* was calculated as (*A. castellanii* infected with *L. pneumophila*/*A. castellanii* concentration of positive control well) × 100%. Then, the percentage of killed *A. castellanii* was measured as (100%—the percentage of survived *A. castellanii*).

*A. castellanii* was cultured in proteose peptone-yeast extract-glucose extract (PYGE) medium in accordance with ATCC protocols (medium 712) and with the addition of 50 mL of 2 M glucose (filter sterilized) (Sigma, St. Louis, MO, USA). Detailed protocol was previously described [9].

### 2.3. THP-1 Cytotoxicity Assay

The virulence of *L. pneumophila* isolates was assessed by infecting THP-1 macrophages at a MOI of 10 for 24 h as previously described [9,11,14,45]. The relative degree of cytopathogenicity was expressed as percent of inhibition compared to non-infected cells; calculated as (Y = [(K − Y)/K] × 100). As K: mean OD of non-infected cells and Y: OD of infected cells. Bacterial density was assessed by the absorbance at 600 nm with a spectrophotometer Nanocolor Vis (Macherey-Nagel, Düren, Germany). 

Cytotoxicity assays of *L. pneumophila* strains were carried out as previously described [9,11,51]. The detailed assay was previously described [9]. 

### 2.4. Pore-Forming Mediated Cytotoxicity Assay

The ability of *L. pneumophila* to lyse sRBCs was assessed at a multiplicity of infection (MOI) of 25 after 2 h of bacterial-sRBCs contact, as described previously [9,12,52]. The release of hemoglobin from lysed red blood cells was measured by spectrophotometry at 415 nm. Pore forming cytotoxicity was expressed as percentage of hemolysis compared to 100% fully hemolyzed blood cells. 

### 2.5. Statistical Analysis

GraphPad Prism software v8.3.0 (Graph-Pad, San Diego, CA, USA) and Primer7 software (Primer-e, Auckland, New Zealand) were used for statistical analyses. Non-Normalized data were normalized. All tests were applied at a 95% and 99% level of confidence. All groups were normally distributed or normalized according to the Shapiro-Wilk test (*p > 0.05*). Variances were equal between groups at all temperatures (Leven’s test, *p > 0.05*). Then, one-way ANOVA was performed to estimate statistical differences among virulence assays and between *L. pneumophila* genotypes and clonal complexes. Also, independent *t*-Test was performed to estimate differences among the three virulence assays. An agglomerative clustering dendrogram was achieved using the Primer7 software in order to study the similarity between virulence characteristics of *L. pneumophila* strains belonging to different genotypes (Gt4(17), Gt6(18), and Gt10(93)) and clonal complexes (VACC1, VACC2, VACC5 and VACC11). The resemblance matrix was calculated using the Bray-Curtis index of association on the pathogenicity variables of the three different in vitro assays. Associations between MLVA-genotypes and clonal complexes were calculated using the Similarity Profile Analysis (SIMPROF) [53] based on Spearman rank correlation.

## 3. Results 

### 3.1. Virulence Characteristics of L. pneumophila MLVA-8(12) Genotypes

To investigate whether the most abundant *L. pneumophila* genotypes in the West Bank exhibit specific virulence characteristics, 60 *L. pneumophila* isolates from eight hospitals across the West Bank were analyzed for cytotoxicity (due to intracellular replication) against *A. castellanii* and THP-1 macrophages, and for contact-dependent pore formation in sRBCs. The presented comparison had a focus on strains MLVA-8(12) genotypes Gt4(17), Gt6(18) and Gt10(93) (Table 1).

**Figure 1:**
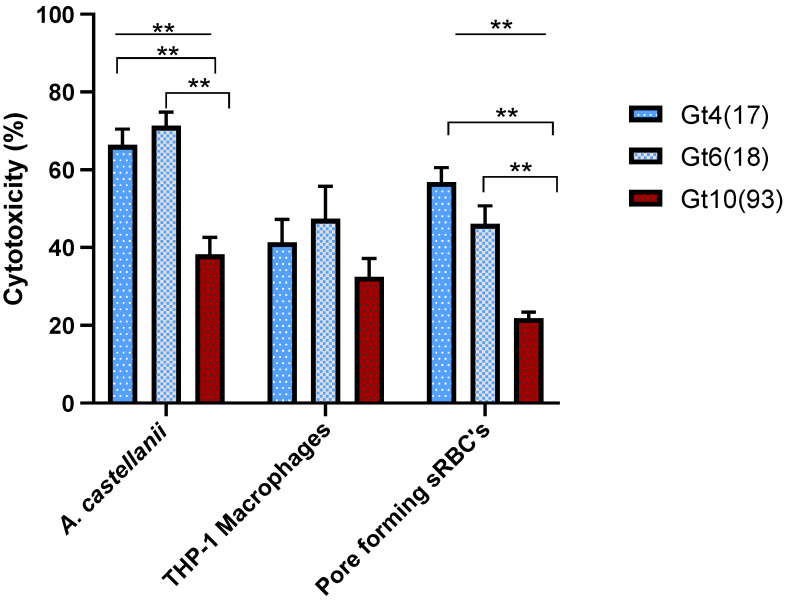
The cytotoxicity of *L. pneumophila* genotypes during post exponential phase was determined by three cytotoxicity tests; infectivity of *A. castellanii* or THP-1 macrophages and pore forming mediated cytotoxicity of sRBCs. *A. castellanii* infection and pore forming cytotoxicity were significantly different (One-Way ANOVA *p* ≤ 0.01) for the genotypes (Line); statistically significant difference (Independent *t*-Test *p* ≤ 0.01) between genotypes appears in Down brackets. Values are means, and error bars represent standard deviations. The * indicates statistically significant differences. ** *p* ≤ 0.01.

Compared to Gt4(17) and Gt6(18) affiliated to ST1, genotype Gt10(93) affiliated to ST461 exhibited the lowest cytotoxicity for *A. castellanii* infection and the lowest contact-dependent pore formation for sRBC´s (38.23 ± 4.38% and 21.8 ± 1.6% respectively) (Table 1 and Figure 1). Since the data are statistically significant (One- Way ANOVA *p* ≤ 0.01), these results demonstrate that Gt10(93) (ST461) has a lower overall virulence in comparison with Gt4(17) and Gt6(18) which are globally prevalent. The virulence did not differ significantly between Gt4(17) and Gt6(18) isolates within all cytotoxicity tests (Figure 1). The non-virulent dotA mutant resulted in less than 15% cell death (Supplementary Material Table S1). A complete list of all isolates analyzed for virulence is provided in Supplementary Material Table S1.

The clinical reference strain *L. pneumophila* str. Paris (Gt 4(17), VACC1, ST1) was tested *by* the previously mentioned cytotoxicity assays. This reference strain had a comparable virulence pattern compared to the set of Gt4(17) strains from the West Bank (cytotoxicity for *A. castellanii*, THP-1 macrophages and contact-dependent pore formation for sRBC´s 62.5 ± 5.9%, 56.8% ± 3.7 and 74.7 ± 4.9% respectively) (Supplementary Material Figure S1 and Supplementary Material Table S1). 

### 3.2. Virulence Characteristics of L. pneumophila Clonal Complexes 

**Figure 2:**
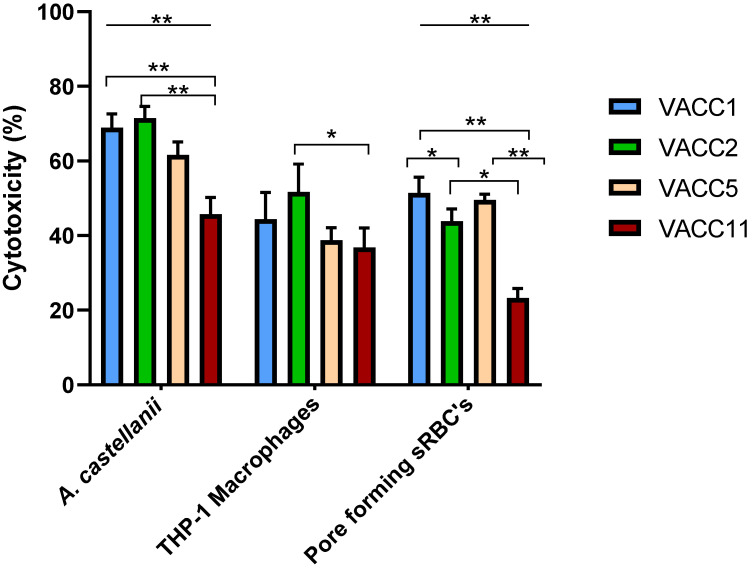
The cytotoxicity of *L. pneumophila* clonal complexes during post exponential phase was determined by three cytotoxicity tests; infectivity of *A.castellanii* or THP-1 macrophages and pore forming mediated cytotoxicity of sRBCs. *A. castellanii* infection and pore forming cytotoxicity are significantly different (One-Way ANOVA *p* ≤ 0.01) for the VACCs (Line); statistically significant difference (Independent *t*-Test *p* ≤ 0.01) between VACCs is displayed in down brackets. Values are means, and error bars represent standard deviations. * indicates statistically significant differences. ** *p* ≤ 0.01 and * *p* ≤ 0.05.

The MLVA-8(12) genotypes sampled from biofilms in the West Bank cluster into four different clonal complexes (VACC1,2,5,11) [38]. To investigate whether the different VACCs correlate with respective virulence potentials of *L. pneumophila*, 60 environmental isolates were analyzed for cytotoxicity against *A. castellanii* and THP-1 macrophages, and for contact-dependent pore formation in sRBCs. The virulence analyses revealed statistically significant (ANOVA *p ≤* 0.01) difference between the four VACCs with respect to cytotoxicity against *A. castellanii,* THP-1 macrophages and pore forming activity in sRBCs (Figure 2).

In terms of virulence against *A. castellanii*, VACC1 and VACC2 showed the highest activities. In terms of significance (Independent *t*-Test *p ≤* 0.05), the activity of VACC1 and VACC2 was significantly higher than the activity of VACC11.

In terms of cytotoxicity against macrophages showed VACC2 the highest activity; however, only the activity of VACC11 was significantly lower than VACC2. 

In terms of pore forming activity in sRBCs, VACC1 and VACC5 showed the highest activities at a comparable level; by far the lowest activities were observed for VACC11. As a negative control, the non-virulent *dotA* mutant resulted in less than 15% cell death (Supplementary Material Figure S1). Detailed data for individual isolates are provided in Supplementary Material Table S1. 

The newly identified clonal complex in the West Bank VACC11 and its genotype Gt10(93) seemed to be significantly less cytotoxic towards amoebae at 37 °C (after a 24-h infection). Also, cytopathogenicity against THP-1 macrophages and hemolytic activity were significantly less compared to VACC1 and its genotypes Gt4(17) and Gt6(18), and in comparison to VACC2 and VACC5 (Figure 2). 

The reference strains *L. pneumophila* str. Paris and *L. pneumophila* str. Philadelphia-1 (highly virulent strains and associated with LD globally) were tested in our study as reference strains for VACC1 (“Paris lineage”) and VACC2 (“Philadelphia-1 lineage”) respectively. The set of three cytotoxicity assays yielded for *L. pneumophila* str. Paris (cytotoxicity for *A. castellanii*, THP-1 macrophages and contact-dependent pore formation for sRBC´s: 62.5 ± 5.9%, 56.8% ± 3.7 and 74.7 ± 4.9%, respectively) virulence traits comparable to VACC1; by contrast *L. pneumophila* str. Philadelphia-1 yielded lower virulence activities compared to strain Paris and VACC2.(49 ± 4.4%, 48.2% ± 3.8 and 61 ± 1.4%, respectively) (Supplementary Material Figure S1 and Supplementary Material Table S1). 

Taken together, these results showed that VACC1-genotypes had consistently high virulence activities; by contrast the newly identified VACC11 had lower virulence characteristics in comparison with the ubiquitous clonal complexes VACC1, VACC2 and VACC5 (Figure 2). 

### 3.3. Hierarchical Agglomerative Cluster Analysis

**Figure 3:**
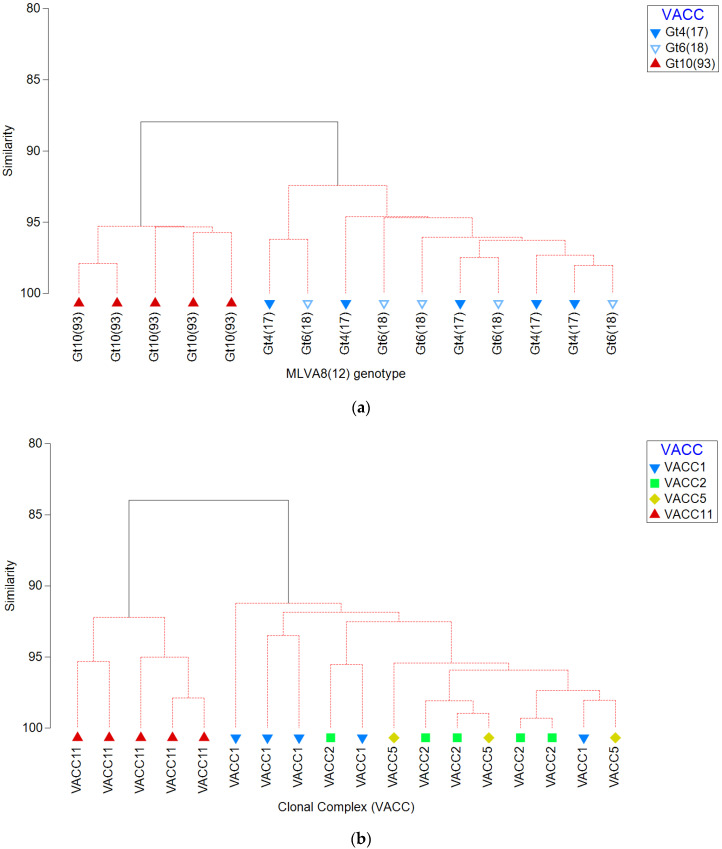
Agglomerative clustering dendrogram representing the percentage of similarity between virulence characteristics of *L. pneumophila* isolates belonging to different (**a**) genotypes (Gt4(17), Gt6(18), and Gt10(93)) and (**b**) clonal complex (VACC1, VACC2, VACC5, and VACC11). The resemblance matrix was calculated using the Bray-Curtis index of association on cytotoxicity characteristics variables, i.e., cytotoxicity against *A. castellanii* and THP-1 macrophages, and pore forming mediated cytotoxicity.

Significant differences were detected between the virulence characteristics of environmental isolates belonging to different genotypes and clonal complexes (Figure 1 and Figure 2). Gt10(93) isolates were significantly less cytotoxic toward *A. castellanii*, THP-1 macrophages and sRBC´s than Gt4(17) and Gt6(17) isolates. Furthermore, VACC11 presented a unique virulence profile with respect to cytotoxicity toward *A. castellanii*, THP-1 macrophages and sRBC´s (Figure 2). Hierarchical agglomerative cluster analysis revealed that isolates belonging to the Gt10(93) and VACC11 clustered together almost homogenously with a genotype and clonal complex-dependent virulence profile (Figure 3). VACC11 (Figure 3b) is abundant in 60% of Gt10(93), 20% of Gt10(141) and 20% of Gt9(92) (Supplementary Material Table S1). This means that not only Gt10(93), which is the most abundant genotype in VACC11, but also the other genotypes in the same clonal complex had comparable virulence characteristics (Supplementary Material Figure S2). This suggests that VACC11 genotypes seem to be less virulent according to the tested traits. The non-VACC11 genotypes and clonal complexes did not form specific clusters according to their genotypes and cluster. Thus, strains affiliated with genotypes of VACC 1, 2 and 5 could not be characterized by a distinguished set of virulence traits, in contrast to VACC11.

## 4. Discussion

There is a wealth of evidence that climate change is with a significant impact on the water resources in the West Bank [54]. Among many waterborne pathogens, *L. pneumophila* can be expected to benefit from elevated temperatures in natural and man-made water systems [43,55]. Therefore, studies on ecology and virulence of *L. pneumophila* are a crucial basis for future management of DWDS.

To elucidate specific infection routes of *L. pneumophila* from the environment to the human population, tracking of genotypes and clonal complexes have proven useful [38,43]. However, for a better preparedness, a combined knowledge of *L. pneumophila* ecology, transmission and virulence is needed. In the past, the degree of *L. pneumophila* virulence was successfully determined by analyzing the infectivity and cytopathogenicity for *A. castellanii* or macrophage-like cells. Other approaches included the induction of pore-formation mediated cytotoxicity of host cells [9,10,26,56,57]. 

In the present study, we combined *L. pneumophila* genotyping with these approaches and were able to show that isolates belonging to different *L. pneumophila* genotypes and VACCs can differ markedly in virulence (Figure 1 and Figure 2). Altogether, 60 environmental *L. pneumophila* isolates from the most abundant genotypes (Gt4(17), Gt6(18) and Gt10(93)) and the clonal complexes VACC1, VACC2, VACC5, and VACC11 were analyzed with respect to cytotoxicity for *A. castellanii*, THP-1 macrophages and pore-forming mediated cytotoxicity in sRBCs. Significant differences were observed between *L. pneumophila* genotypes and VACCs. *L. pneumophila* Gt4(17) and Gt6(18) affiliated with VACC1 and ST1, isolated from six hospitals (A-F) and hospital G, respectively, showed the highest virulence (Figure 1). These results are in accordance with a previous study [58] demonstrating that ST1 strains of *L. pneumophila* can be highly virulent, in addition to their worldwide high prevalence. Genotypes Gt4(17) and Gt6(18) are affiliated with ST1 and VACC1, the most abundant clonal complex and sequence type in the West Bank and worldwide. VACC11 is a clonal complex identified for the first time in the West Bank by Zayed et al. [38]. This clonal complex exhibits a comparably low virulence potential (Figure 2). 

According to epidemiological studies worldwide [59,60] ST1 (includes Gt4(17) and Gt6(18)) is one of the most virulent ST that have been described for *L. pneumophila* strains since it is one of the most frequent cause of LD globally; observations of this study suggest that this may also be valid for the West Bank.

Agglomerative clustering dendrograms revealed that virulence profiles for genotypes were rather different between the relevant VACC11-genotype Gt10(93) on one hand, and on the relevant VACC1-genotypes (Gt4(17) and Gt6(18)) on the other hand, in the West Bank (Figure 3). This discrepancy was also observed when comparing a larger set of genotypes: VACC11-genotypes clustered together and separate from the other clonal complexes. Figure 3 also shows a distinct cluster for VACC11, but a mixed cluster for all genotypes of the other clonal complexes VACC1, 2 and 5. 

Our results demonstrated that there was no significant difference in the cytotoxicity toward THP-1macrophages between the genotypes and VACCs of *L. pneumophila* strains except between VACC2 and VACC11. However, a statistically significant difference was seen between *L. pneumophila* genotypes and VACCs for *A. castellanii* infection and pore forming cytotoxicity. An explanation might be that environmental isolates can be expected to have more pronounced genotype-dependent environmentally relevant defense traits. 

The resolution of MLVA-8(12) genotyping applied in this study for *L. pneumophila* strains allowed the classification and comparison of the pathogenicity potential of each genotype in order to determine which genotype poses the greatest risk to public health. MLVA-8(12) is very useful because it allows classification within the highly health-relevant and globally distributed ST1, comprising the reference strain *L. pneumophila* strain Paris [41]. The classical SBT does not distinguish below the level of ST1. However, especially for this globally highly relevant sequence type a higher distinction is needed.

Strains of VACC11 include three genotypes (Gt10(93), Gt10(141) and Gt9(92)), are affiliated with ST461, and were classified as Sg 6 Dresden [38]. ST461 was previously reported by the European Working Group for *Legionella* infections (EWGLI) [30,38,57,61], to be found in hospitals water in Poland [61]. Recently, ST461 was identified in Michigan, (USA) water systems and showed high capability to efficiently infect THP-1 macrophages [57]. More recently, ST461 was identified in hotel water in southern Israel [30] and in the West Bank hospital water systems [38]. 

*L. pneumophia* from clinical and environmental samples, collected from suspected pneumonia patients and from biofilm samples of different wards in one hospital in East Jerusalem. *L. pneumophila* was detected in 35% of Bronchoalveolar lavage (BAL) samples and 15% of sputum samples using conventional PCR. By using Nested PCR sequence-based typing (NPSBT), 29% of clinical samples genotyped ST1 and 21% genotyped ST461. Jaber et al. [32] findings support our idea that the information of environmental samples in combination with genotyping at high resolution level (as MLVA-8(12)) are needed to understand the epidemiology of legionellosis in a certain geographical area. 

A study by Sharaby et al. [9] analyzing clinical and environmental *L. pneumophila* isolates from northern Israel, showed that Gt4 virulence are in concordance with our results with respect to a high cytotoxicity towards *A. castellanii* and THP-1 macrophages. Half of the Israeli clinical isolates and a major fraction of the environmental strains were Gt4 and Gt6 (both affiliated with ST1 and VACC1) indicating the clinical importance and environmental relevance of these genotypes and ST1 also in DWDS of Israel. The corner stone between our study and Sharaby et al. is a “genotype-site specificity”, i.e., specific genotypic groups occurring in a DWDS in Oranim campus (Haifa city) [9,37], had specific ecotype characteristics (i.e., describing a set of strains of *L. pneumophila* inhabiting a specific niche) [44], comparable to our findings in the West Bank [38,43]. Therefore, “genotype-site specificity” for *L. pneumophila* strains may include a set of genotype-dependent specific traits, such as pathogenicity, virulence, epidemiologically relevant and ecological characteristics with a specific local “imprint”.

A study by Sousa et al. [62] looked at differences between *L. pneumophila* isolates from clinical, man-made and natural environmental samples in *Galleria mellonella* infection models in which they found that all strains proved to be pathogenic. They observed that some of the strains were defective in their ability to cause disease while others were highly pathogenic. Sousa et al. [62] concluded from their findings that some *L. pneumophila* strains’ ability to cause disease is more related to their capability to persist and bloom in man-made environmental niches, which kind of mimics human infection, and less dependent on their virulence [62,63]. This observation is of relevance because environmental *L. pneumophila* strains are reservoirs and source of sporadic cases of legionellosis [64,65]. Another important aspect is how *L. pneumophila* strains behave in the environment, select their niche or co-inhabit niches as genotype consortia, and how this affects the virulence of specific genotypes. Both biotic and abiotic factors may influence structure and dynamics of *L. pneumophila* populations as shown by many authors in DWDS and more specifically for the West Bank [37,43,66]. Though, it is well known that amoeba are an important training ground for *L. pneumophila*’s pathogenicity, little is known of the influence of other biotic and abiotic factors, including interactions among different genotypes on the resulting virulence of *L. pneumophila* [11,17]. 

### 4.1. Relevance of the Findings for the West Bank and Beyond

Jaber et al. [32] showed that there was a high risk of lung infection in the West Bank due to *L. pneumophila* as indicated by the high percentage of infected pneumonia patients. Furthermore, they have shown that the sequence types most relevant for *L. pneumophila* caused pneumonia were ST1 (29%) and ST461 (21%).

By high resolution MLVA-8(12) analysis [38,41], these sequence types could be resolved into a set of genotypes. As shown by regional and abundance analysis in the West Bank, the most relevant representatives in the West Bank for ST1 were the VACC1-MLVA-8(12-genotypes Gt4(17) and Gt6(18), and for ST461 the VACC11-MLVA-8(12) genotypes Gt10(93), Gt10(141), and Gt9(92). While the ST1-genotypes played an eminent role also in Israel, the ST461-genotypes were only observed in the West Bank.

Relevance for LD can be assumed for the ST1-genotypes due to their virulence traits. Less expected is the important role of the ST461-genotypes for LD in the West Bank due to their lower virulence profile. However, virulence traits and the overall infection processes are rather complex phenomenons. In addition, there are other aspects contributing to the risk of LD, such as the infective dose (see Quantitative Microbial Risk Assessment (QMRA) below). 

Another aspect for risk of *L. pneumophila* infections is its complex ecology leading to a rather different concentration of *L. pneumophila* in DWDS. As shown in previous studies [36,37,38,45], *L. pneumophila* displayed a genotype-dependent preference with respect to habitat characteristics (ecotype), leading to a specific genotype pattern and abundance in water and biofilm. Since infection is not only dependent on virulence traits but also on the infective dose, the ecology exerts a direct impact on the infection risk. QMRA addresses this risk as a well-established tool [67]. The genotype-dependence of ecology and virulence of this study and previous ones in the West Bank and Israel [9,36,37,38,45] suggest that modeling of abundance and risk (QMRA) might be more precise when applied on the genotype level of *L. pneumophila.* MLVA-8(12)-genotyping may provide a good level of resolution to address these issues.

Source tracking based on the MLVA-genotypes could be furthermore helpful to assess the sources of infection in the West Bank and of great value for risk management. 

### 4.2. Conclusions and Future Research

This is the first study in which virulence characteristics of environmental *L. pneumophila* isolates from the West Bank were compared. Overall, we observed clustering of specific genotypes and VACCs with specific virulence potentials. The presented results suggest that information on virulence characteristics in combination with genotyping at an adequate resolution level (such as MLVA) is helpful to improve public health management and risk assessment measures. 

Most of the *L. pneumophila* isolates were retrieved from the biofilm of the DWDS in the West Bank. In this set of isolates no significant difference in terms of virulence between strains derived from biofilm and bulk water could be assessed, in consistence with a previous study from Israel [9]. However, exchange processes between biofilm and water for *L. pneumophila* are complex and would need more detailed investigation on the genotype level. The genotype-dependent influence of Magnesium on *L. pneumophila* abundance may play in this respect a special role in the West Bank, and may be worth a closer look also for other geographic and climatic regions.

## Figures and Tables

**Table 1:** *Legionella pneumophila* genotypes used in this study ¹.

Strain Characteristics	MLVA-8(12) Genotype (Gt)	Clonal Complex (VACC)	Sequence Type (ST)	Serogroup (Sg), Mab ²	Sampling Site (Hospital)	No. of Isolates
	Gt4(17)	VACC1	ST1	Sg1	A-F	12 ˟
Environmental (Biofilm and Water isolates)	Gt6(18)	VACC1	ST1	Sg1	G	13 ˟
	Gt10(93)	VACC11	ST461	Sg6 Dresden	F	12 °
	DT ³	VACC2	DT ³	Sg6 Dresden, Sg10	A, D, E, F	11
	DT ³	VACC5	DT ³	Sg6 Dresden *	A, G, H	5
	DT ³	VACC11	ST461	Sg6 Dresden	B, F	7 °
Reference strains						
Paris	Gt4(17)	VACC1	ST1	Sg1	Clinical sample	
Philadelphia-1	Gt64(74)	VACC2	ST36	Sg1	Clinical sample	
*dotA* mutant	*L. pneumohila* Philadelphia-1 *icm/dot*-defient mutant strain (negative control)					

¹ For more details, see supplementary materials (Supplementary Material Table S1); ² MAb: Monoclonal Antibody; ³ DT: Different Types; ˟ Total No. of VACC1 isolates is 12 + 13 = 25 isolates; ° Total No. of VACC11 isolates is 12 + 7 = 19 isolates; * A166 is not typed by Mab. Sg (2–14) (Details Supplementary Material Table S1)

## Data Availability

Data is contained within the article or Supplementary Material.

## Supplementary Materials

The following are available online at https://www.mdpi.com/article/10.3390/pathogens10040417/s1, Figure S1: The virulent activity of *L. pneumophila* reference strains (*L. pneumophila* str. Paris, *L. Pneumophila* str. Philadelphia-1 and its Icm/Dot deficient *dotA* mutant as a negative control) during post exponential phase was determined by three cytotoxicity tests; cytotoxicity against *A. castellanii* or THP-1 macrophages and pore forming mediated cytotoxicity of sRBC’s, Figure S2: Agglomerative clustering dendrogram representing the percentage of similarity between cytotoxicity characteristics of *L. pneumophila* isolates belonging to different genotypes (Gt4(17), Gt6(18), Gt10(93), Gt10(141), and Gt9(92)). The resemblance matrix was calculated using the Bray-Curtis index of association on cytotoxicity characteristics variables against *A. castellanii* cytotoxicity, cytopathogenicity of THP-1 macrophages and pore forming mediated cytotoxicity, Table S1: List of *L. pneumophila* strains (*n* = 60) isolated from the West Bank analyzed in this study by different in vitro cytotoxicity tests. *L. pneumophila* reference strains included. Water isolates are highlighted in bold.
