# Supplementary material for: Cytotoxicity, Intracellular Replication, and Contact-Dependent Pore Formation of Genotyped Environmental Legionella pneumophila Isolates from Hospital Water Systems in the West Bank, Palestine
Source: Pathogens. 2021 Apr 1;10(4):417. doi: 10.3390/pathogens10040417 (PMC8066006; doi:10.3390/pathogens10040417)
Supplement: Supplementary file 1 [file pathogens-10-00417-s001.pdf]

Supplementary Materials

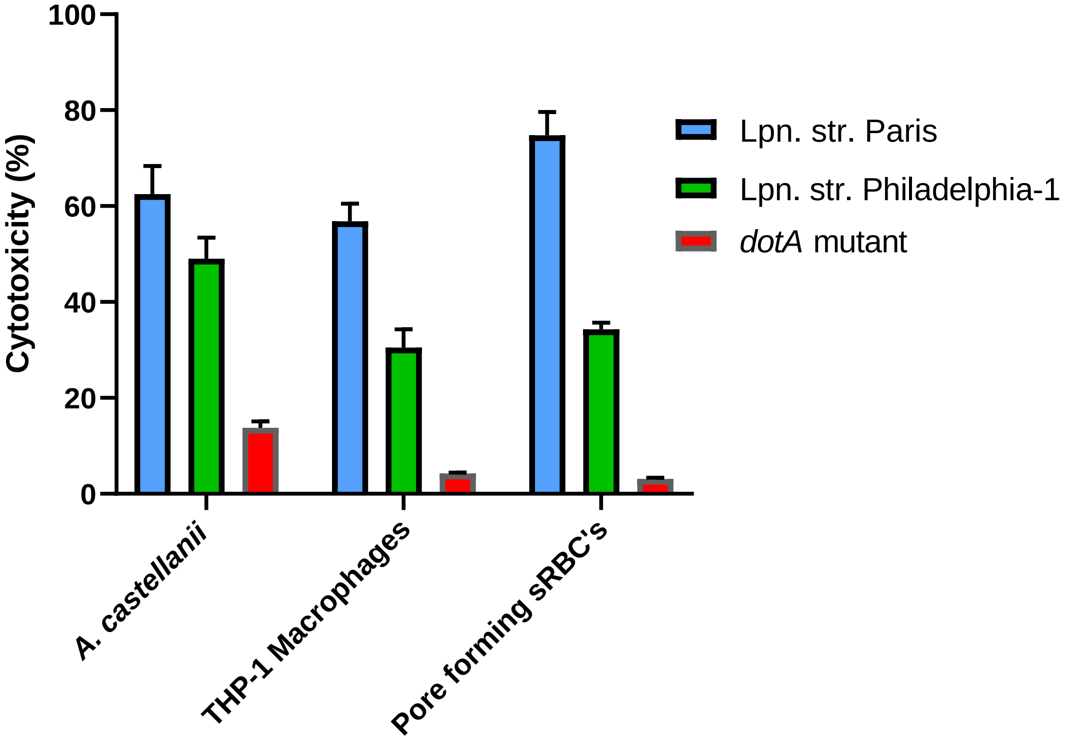

Figure S1. The virulent activity of *L. pneumophila* reference strains (*L. pneumophila* str. Paris, *L. Pneumophila* str. Philadelphia-1 and its Icm/Dot deficient *dotA* mutant as a negative control) during post exponential phase was determined by three cytotoxicity tests; cytotoxicity against *A.castellanii* or THP-1 macrophages and pore forming mediated cytotoxicity of sRBC's.

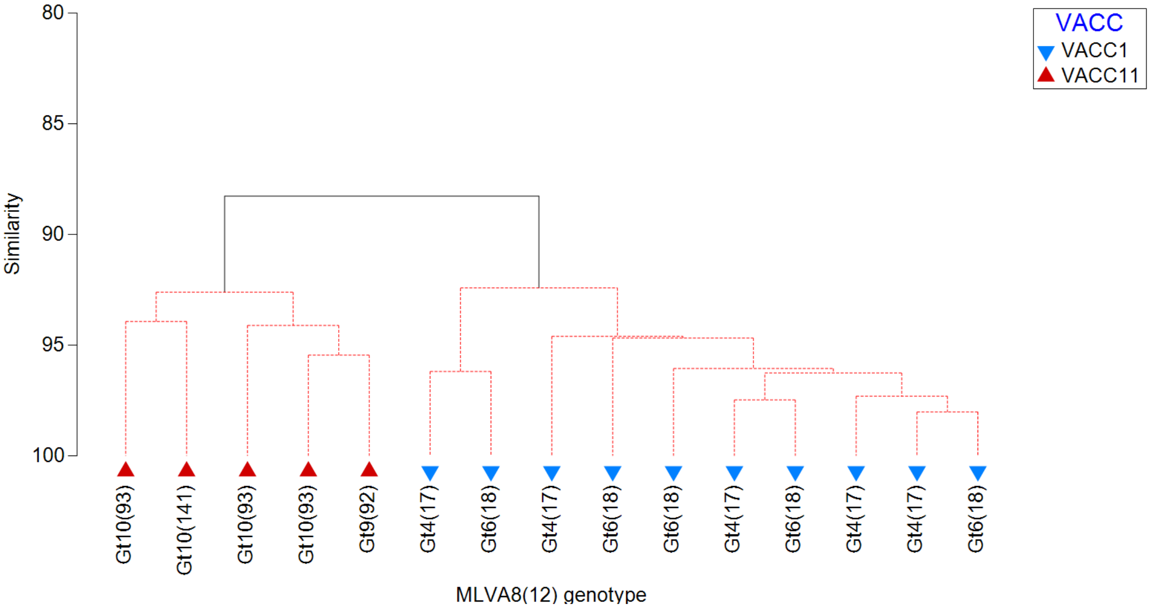

Figure S2. Agglomerative clustering dendrogram representing the percentage of similarity between cytotoxicity characteristics of *L. pneumophila* isolates belonging to different genotypes (Gt4(17), Gt6(18), Gt10(93), Gt10(141), and Gt9(92)). The resemblance matrix was calculated using the Bray-Curtis index of association on cytotoxicity characteristics

variables against *A. castellanii* cytotoxicity, cytopathogenicity of THP-1 macrophages and pore forming mediated cytotoxicity.

Table S1. List of *L. pneumophila* strains (n=60) isolated from the West Bank analyzed in this study by different in vitro cytotoxicity tests. *L. pneumophila* reference strains included. Water isolates are highlighted in bold.

Isolate Designation	Gt	Sg	VACC	Hospital	ST	Pore forming sRBC's(%)	Std (%)	THP-1 (%)	Std (%)	*A. castellanii*(%)	Std (%)
A1	Gt4(17)	1	1	D	ST1	57.25	8.11	37.03	9.68	74.30	3.20
A104	Gt4(17)	1	1	B	ST1	70.62	4.84	47.22	20.86	64.60	2.70
A109	Gt4(17)	1	1	F	ST1	51.59	2.38	37.36	3.00	64.60	3.10
A122	Gt4(17)	1	1	D	ST1	45.77	3.46	47.02	4.94	72.20	5.70
A137	Gt4(17)	1	1	B	ST1	67.44	5.52	50.07	5.12	58.87	3.90
A139	Gt4(17)	1	1	B	ST1	68.56	5.76	16.67	1.85	67.57	0.00
A141	Gt4(17)	1	1	B	ST1	74.50	1.69	45.38	4.01	56.68	6.54
A142	Gt4(17)	1	1	E	ST1	63.52	5.53	9.23	3.21	63.58	7.69
A170	Gt4(17)	1	1	A	ST1	39.35	1.57	41.67	3.40	71.43	5.83
A171	Gt4(17)	1	1	A	ST1	74.50	1.69	45.38	4.01	59.52	3.37
A172	Gt4(17)	1	1	A	ST1	27.80	0.81	54.68	7.08	69.05	3.37
A176	Gt4(17)	1	1	C	ST1	41.00	3.32	64.33	4.28	74.36	3.63
A25	Gt6(18)	1	1	G	ST1	56.49	1.17	48.40	4.03	68.74	1.80
A29	Gt6(18)	1	1	G	ST1	20.80	1.42	39.35	1.34	59.46	0.00
A130	Gt6(18)	1	1	G	ST1	28.39	3.34	32.16	1.04	73.35	3.25
A131	Gt6(18)	1	1	G	ST1	67.95	17.93	60.04	10.58	89.74	3.63
A133	Gt6(18)	1	1	G	ST1	73.66	14.27	34.44	1.01	71.69	3.65
A135	Gt6(18)	1	1	G	ST1	23.34	3.80	35.35	5.99	64.29	5.83
A186	Gt6(18)	1	1	G	ST1	27.56	0.74	93.14	14.08	87.18	3.63
A187	Gt6(18)	1	1	G	ST1	32.29	1.04	18.05	9.14	80.58	2.21
A188	Gt6(18)	1	1	G	ST1	57.52	3.42	15.60	8.15	56.85	6.65
A189	Gt6(18)	1	1	G	ST1	52.65	2.44	85.42	26.12	58.65	6.54
A190	Gt6(18)	1	1	G	ST1	49.24	2.64	38.63	8.37	74.36	3.56
A191	Gt6(18)	1	1	G	ST1	52.33	4.55	59.06	7.06	74.20	3.68
A192	Gt6(18)	1	1	G	ST1	56.44	4.31	56.68	12.08	66.68	0.68
A44	Gt13(72)	6	2	A	ST1326	49.95	2.47	41.89	1.82	67.63	2.68
A69	Gt13(72)	6	2	D	ST1326	42.02	3.08	34.23	11.37	65.71	3.64
A70	Gt13(72)	6	2	D	ST1326	51.78	3.28	51.46	6.17	64.80	5.36
A95	Gt13(72)	6	2	E	ST1326	69.96	2.47	41.89	1.82	67.65	3.55
A98	Gt13(72)	6	2	F	ST1326	21.78	2.28	61.46	6.17	75.36	3.25
A119	Gt64(74)	6	2	F	ST9	18.53	0.68	88.64	24.10	74.68	1.98
A129	Gt64(74)	6	2	F	ST9	18.62	3.82	49.97	4.58	83.78	0.00
A144	Gt13(72)	6	2	E	ST1326	42.04	3.05	34.13	11.35	65.68	3.60
A156	Gt64(74)	6	2	F	ST9	44.03	4.77	57.23	7.18	67.57	6.62
A196	Gt 13(143)	10	2	E	ST1326	65.37	3.80	34.44	1.09	74.63	4.20
A197	Gt 13(143)	10	2	E	ST1326	58.33	6.66	73.32	6.72	73.37	1.14
A46	Gt 16(1)	6	5	A	ST1438	36.97	1.61	39.87	4.57	63.55	2.47

A166	Gt 8(142)	(2-14)	5	G	ST1482	48.95	0.73	49.97	4.58	67.32	2.36
A193	Gt 40(47)	6	5	H	ST292	52.87	0.83	34.67	3.39	80.65	0.00
A194	Gt 40(47)	6	5	H	ST292	50.92	2.41	40.83	3.49	83.87	4.56
A195	Gt 40(47)	6	5	H	ST292	58.17	1.87	28.49	0.61	12.90	7.90
A99	Gt10(93)	6	11	F	ST461	28.50	1.94	36.13	12.88	40.50	3.37
A108	Gt10(93)	6	11	F	ST461	37.89	2.26	25.91	3.55	59.57	3.01
A112	Gt10(93)	6	11	F	ST461	26.80	2.48	23.24	8.62	27.66	3.01
**A114**	**Gt10(93)**	**6**	**11**	**F**	**ST461**	**13.06**	**0.79**	**29.68**	**2.90**	**48.94**	**5.21**
A115	Gt10(93)	6	11	F	ST461	22.58	0.95	41.65	1.60	29.40	4.30
A127	Gt10(93)	6	11	F	ST461	14.45	1.00	37.53	3.31	44.68	6.02
A128	Gt10(93)	6	11	F	ST461	34.80	3.70	12.94	1.36	38.60	3.37
A152	Gt10(93)	6	11	F	ST461	17.83	0.42	25.93	6.07	36.50	5.60
A153	Gt10(93)	6	11	F	ST461	14.45	1.00	35.01	1.25	25.60	3.90
A182	Gt10(93)	6	11	F	ST461	14.88	2.02	37.42	5.73	37.50	3.80
A183	Gt10(93)	6	11	F	ST461	16.78	0.70	40.58	3.28	42.60	7.90
A198	Gt10(93)	6	11	F	ST461	19.53	2.00	43.66	5.70	27.20	3.05
A116	Gt10(141)	6	11	F	ST461	25.79	5.22	48.47	7.58	74.19	4.56
A138	Gt9(92)	6	11	B	ST461	30.94	6.97	37.35	6.54	25.60	3.68
**A148**	**Gt10(141)**	**6**	**11**	**F**	**ST461**	**19.53**	**2.00**	**39.62**	**5.70**	**76.92**	**6.28**
A149	Gt10(141)	6	11	F	ST461	12.92	3.71	39.98	6.47	64.62	0.00
A157	Gt10(141)	6	11	F	ST461	18.87	3.25	42.40	5.56	70.65	7.90
A177	Gt9(92)	6	11	B	ST461	30.47	1.44	41.08	4.36	27.36	5.87
A178	Gt9(92)	6	11	B	ST461	35.19	1.40	39.35	3.94	32.69	4.36
Lpn str. Paris	Gt4(17)	1	1		ST1	74.70	4.90	56.80	3.70	62.50	5.90
Lpn str. Philadelphia-1	Gt64(74)	1	2		ST36	34.30	1.30	30.50	3.80	49.00	4.40
dotA mutant						3.10	0.25	4.20	0.25	13.70	1.40
